# Implementing Self-Regulated Learning in Classrooms: Connecting What Primary School Teachers Think and Do Through Video-Based Observations and Interviews

**DOI:** 10.3390/bs15121627

**Published:** 2025-11-26

**Authors:** Lies Backers, Hilde Van Keer

**Affiliations:** Department of Educational Studies, Faculty of Psychology and Educational Sciences, Ghent University, 9000 Ghent, Belgium; hilde.vankeer@ugent.be

**Keywords:** self-regulated learning, primary school teachers, teacher knowledge, teacher beliefs, teacher practice, observation, video-stimulated reflection interview

## Abstract

Self-regulated learning (SRL) is crucial for effective learning, supporting academic achievement and lifelong competencies. Fostering SRL in primary education is important, yet teachers’ understanding and use of strategies are underexplored. This study provides an innovative, multi-method investigation of whether and how primary school teachers’ knowledge and beliefs about SRL align with their classroom practices. Video-based classroom observations were combined with semi-structured interviews to capture both what teachers think and what they do. The study addressed three research questions: (1) how and to what extent teachers implement SRL; (2) their knowledge and beliefs regarding SRL and alignment of these with classroom practice; (3) factors perceived as facilitating or constraining SRL implementation. Eight teachers participated, providing 16 h of observations and 11 h of interview data. Observations were analyzed using the ATES instrument, and interviews were coded thematically. Findings revealed variation in SRL implementation and misalignments between knowledge, beliefs, and practice. Teachers held misconceptions and focused mainly on metacognitive and motivational strategies in classroom practice. Limited self-efficacy and school- and classroom-level factors further constrained SRL implementation. Results indicate a need for professional development addressing knowledge gaps, misconceptions, and teachers’ self-efficacy, while encouraging school-wide reflective practices to support SRL in primary classrooms.

## 1. Introduction

In today’s rapidly evolving society, students are expected not only to acquire knowledge but also to manage and regulate their own learning in increasingly autonomous ways. In this context, self-regulated learning (SRL) plays a critical role in achieving academic success and fostering effective lifelong learning (Dent & Koenka, 2016). SRL is a multifaceted and demanding process that integrates metacognitive skills (e.g., planning, goal setting, monitoring, and self-evaluation), cognitive strategies (e.g., selecting learning techniques, organizing learning environments), and motivational components (e.g., self-efficacy, interest, and attribution patterns) (Zimmerman, 2002). However, SRL does not develop spontaneously: students require guidance and opportunities to acquire these skills. Teachers play a pivotal role in supporting this development, both directly, by explicitly teaching learning strategies, and indirectly, by creating classroom environments that foster autonomy, motivation, and collaborative engagement (Perry et al., 2004). Although the importance of SRL is widely acknowledged, prior research suggests that teachers often face challenges in implementing SRL due to limited or fragmented knowledge, misconceptions about the SRL concept, or low self-efficacy beliefs regarding its instruction (De Smul et al., 2018; Dignath & Büttner, 2018; Spruce & Bol, 2015). Moreover, while teachers may endorse the idea of fostering SRL, this does not always translate into intentional or explicit practices in their classroom (Dignath-van Ewijk et al., 2013). Although studies indicate that a gap exists between what teachers do in the classroom and what they know and believe about promoting SRL, findings on the relationship between aspects of teachers’ competences regarding SRL (e.g., knowledge and beliefs) and their self-reported or observed SRL implementation remain inconsistent (Karlen et al., 2020; Kramarski & Heaysman, 2021; Spruce & Bol, 2015; Wilson & Bai, 2010). As a result, our current understanding of what teachers do in authentic classroom situations to support SRL, how this aligns with their knowledge and beliefs regarding SRL and where and why discrepancies arise is still limited.

To address this gap in our knowledge, the present study adopts a mixed-methods approach. We systematically capture teachers’ classroom practices through video-based classroom observations, focusing specifically on how they promote SRL in authentic teaching situations. Based on these videos, we then conduct semi-structured interviews to explore teachers’ thinking and their underlying knowledge, beliefs, and misconceptions regarding SRL. This dual approach offers an integrated, innovative perspective. It allows us to examine not only what teachers do, but also why they do it, thereby uncovering potential alignments or discrepancies between their observed SRL practices and their professional thinking. In doing so, the study aims to provide an integrated understanding of how SRL is conceptualized and enacted by primary school teachers.

## 2. Theoretical Background

### 2.1. Self-Regulated Learning

Self-regulated learning (SRL) is broadly defined as the process through which learners take active control over their learning by setting goals, selecting and applying strategies, monitoring progress, and reflecting on outcomes (Zimmerman, 2002). Over recent decades, multiple theoretical frameworks have enhanced our understanding of SRL, with broad consensus that effective self-regulated learners intentionally regulate not only their cognition but also their motivation, emotions, and behaviors to achieve their objectives (Boekaerts, 1999; Efklides, 2011; Zimmerman, 2002).

Among the different theoretical frameworks, Boekaerts’ (1999) three-layer model identifies three interacting domains of strategies operating within the information-processing cycle: cognitive strategies, which directly influence the processing and retention of information; metacognitive strategies, which indirectly shape learning by initiating, sustaining, and monitoring the learning process; and motivational strategies, which are closely tied to emotions and help maintain effort and persistence. Each domain comprises a range of more specific sub-strategies that, when effectively coordinated, enable learners to adapt their approach to varying task demands (Boekaerts, 1999).

Although meta-analyses consistently show that SRL is positively associated with academic performance, motivation, and engagement across diverse student populations (Dent & Koenka, 2016; Dignath & Büttner, 2008; Elhusseini et al., 2022), it remains an effortful and cognitively demanding process. Students with limited prior experience in self-regulation often struggle to coordinate multiple strategies under substantial cognitive load, and SRL skills rarely emerge spontaneously (Perry et al., 2004; Schuster et al., 2020). This underscores the critical, dual role of teachers: creating learning environments that foster SRL and explicitly teaching the strategies students need to regulate their own learning (Dignath-van Ewijk, 2016). Yet, evidence suggests that teachers, like their students, frequently face challenges in understanding and implementing SRL effectively (Dignath & Büttner, 2018; Spruce & Bol, 2015).

### 2.2. What Teachers Do: Direct and Indirect Promotion of SRL

Instructional practices to promote SRL can be broadly categorized as direct or indirect approaches (Dignath & Veenman, 2020; Dignath-van Ewijk et al., 2013). Direct promotion involves teaching of learning strategies, modeling of regulatory behaviors, guided reflection, and scaffolding activities such as planning, monitoring, and evaluation learning processes. Teachers may employ an implicit form of direct promotion by demonstrating the use of a strategy or assigning a task that requires its application, without explicitly labelling it as a learning strategy (Dignath & Veenman, 2020). Alternatively, in explicit direct strategy instruction, teachers clearly explain how, when, and why a specific learning strategy should be applied, enabling students to deliberately integrate it into their repertoire (Dignath & Veenman, 2020; Perry et al., 2004). Indirect promotion, by contrast, focuses on creating learning environments that encourage autonomy, motivation, and engagement, such as offering meaningful choices, setting challenging tasks, fostering a positive emotional climate, and providing opportunities for collaborative learning (Perry et al., 2004). Integrating both direct and indirect approaches to fostering SRL is considered particularly effective for enhancing students’ SRL competences (Kistner et al., 2010; Paris & Paris, 2001). The development of SRL is most successful when embedded within the teaching of subject matter, rather than treated as an extracurricular or separate activity.

Much of the existing research on teachers’ SRL promotion has relied dominantly on self-report questionnaires, which constitutes a methodological limitation and restricts our understanding of the actual complex processes occurring in the classroom (Kramarski & Heaysman, 2021). In contrast, classroom observational studies can provide a more nuanced picture, revealing differences in the frequency and quality of these practices. Observational studies consistently show that teachers devote limited instructional time to the direct instruction of learning strategies, and that such instruction is seldom made explicit (Dignath & Büttner, 2018; Dignath & Veenman, 2020; Spruce & Bol, 2015; Vosniadou et al., 2024). When explicit SRL promotion does occur, it is often directed toward cognitive strategies (e.g., summarizing, elaboration), while metacognitive strategies (e.g., planning, monitoring, and evaluation) receive comparatively limited attention (Dignath & Veenman, 2020). Instead, direct SRL promotion frequently takes an implicit form, with teachers modelling strategies or embedding them in tasks without explicitly labelling or explaining them as such (Dignath & Büttner, 2018). Indirect approaches, such as designing autonomy-supportive learning environments, emerge as more common than direct SRL instruction (Dignath & Büttner, 2018; Dignath & Veenman, 2020). In the study by Rosenthal et al. (2024), lower secondary school teachers primarily promoted SRL indirectly by fostering positive student emotions through supportive classroom climates. Similarly, the work of Vosniadou et al. (2024) suggests that the relatively low frequency of constructive and interactive lesson tasks observed in their study with secondary school teachers may indicate that many teachers either do not fully understand or undervalue the importance of designing such environment to enhance student learning and foster the development of SRL. Such findings highlight the complexity of fostering SRL in classrooms, as its successful promotion depends not only on the use of specific instructional methods, but also on teachers’ professional thinking (e.g., their knowledge and beliefs regarding SRL) and their ability to adapt strategies to students’ needs. Examining this is therefore crucial for supporting effective SRL implementation and improving student learning outcomes.

### 2.3. What Teachers Think: Knowledge and Beliefs

#### 2.3.1. Teachers’ Knowledge About SRL

Teachers’ professional knowledge is crucial for promoting SRL (Karlen et al., 2020). This includes content knowledge about SRL (CK-SRL), namely understanding SRL concepts, models, and (motivational, cognitive, metacognitive) strategies, and pedagogical content knowledge about SRL (PCK-SRL). PCK-SRL refers to the knowledge of how to teach these processes using both direct and indirect approaches (Barr & Askell-Williams, 2019; Karlen et al., 2020; Shulman, 1987). Despite its importance, research indicates that teachers’ CK and PCK regarding SRL are frequently limited. CK is often weakest in the area of metacognitive strategies (Barr & Askell-Williams, 2019; Dignath & Büttner, 2018), while PCK for classroom implementation is generally low (Barr & Askell-Williams, 2019; Karlen et al., 2020). The predominant misconception identified in the literature concerns the equation of SRL with independent learning or self-directness (Callan & Shim, 2019; Dignath & Sprenger, 2020; Šimić Šašić et al., 2023; Spruce & Bol, 2015). For example, Šimić Šašić et al. (2023) found that although most teachers had heard of SRL, only a small proportion could define it accurately, and few included strategy instruction in their definitions. Similar patterns have been reported by Spruce and Bol (2015) and Dignath and Sprenger (2020), who note that teachers frequently equate SRL with “learning independently” without recognizing the role of explicit instruction in fostering SRL skills. Such misconceptions may contribute to inconsistent classroom practices (Šimić Šašić et al., 2023).

Moreover, research has identified a persistent gap between teachers’ SRL knowledge and their classroom practice. Wilson and Bai (2010) found only a weak correlation between the two and even teachers with relatively high SRL knowledge did not consistently translate this into instructional practices that promote SRL. Dignath-van Ewijk and van der Werf (2012) similarly found that teachers often focus on fostering autonomy and constructivist learning environments but neglect the direct teaching of learning strategies. This disconnect highlights the need to investigate not only what teachers know about SRL, but whether and how that knowledge is enacted in their teaching.

#### 2.3.2. Teacher Beliefs About SRL

Teacher beliefs about SRL refer to viewing learning as a constructive process and recognizing the importance of SRL for academic achievement (Greene, 2021). Beliefs can play a decisive role in facilitating or constraining changes in teaching practices and the adoption of innovative approaches (Lawson et al., 2019; Lombaerts et al., 2009). Yet, findings in SRL research are inconsistent (Steinbach & Stoeger, 2016). Several studies report a direct, positive relationship between teachers’ SRL beliefs and their self-reported SRL practices (e.g., Dignath-van Ewijk, 2016; Lombaerts et al., 2009). In contrast, Spruce and Bol (2015), who assessed SRL implementation through classroom observations and measured teachers’ beliefs using a questionnaire, found no such link. Additionally, Vosniadou et al. (2021) reported that internal inconsistencies within teachers’ beliefs may also influence their instructional practices. Teachers can simultaneously hold beliefs that align with and contradict SRL theory, and beliefs inconsistent with SRL theory were found to negatively predict SRL-related practices (Vosniadou et al., 2021; Darmawan et al., 2020).

A specific and widely studied type of teacher beliefs concerns their self-efficacy beliefs, which refer to teachers’ individual’s belief in their capacity to organize and execute the actions necessary to achieve desired performance outcomes (Bandura, 1977). In the context of SRL, teachers’ self-efficacy to implement SRL reflects their personal beliefs about their ability to foster SRL in the classroom (De Smul et al., 2018). Research indicates that teachers generally feel moderately to highly competent in implementing direct instruction, providing students with challenging and complex tasks, and incorporating evaluation. However, they report uncertainty when it comes to offering students choices in the learning process (De Smul et al., 2018). Teachers’ self-efficacy beliefs for promoting SRL have been found to be positively and strongly associated with both their self-reported SRL implementation (De Smul et al., 2018; Dignath-van Ewijk, 2016) and their beliefs about SRL (Dignath-van Ewijk, 2016).

### 2.4. The Present Study

The relationship between what teachers know about SRL and how they enact SRL-supportive practices remains unclear. Studies using self-reported data often find positive associations between teachers’ SRL-related beliefs or knowledge and their reported practices (Dignath-van Ewijk & van der Werf, 2012; Lombaerts et al., 2009), but observational studies rarely replicate these findings (Dignath & Veenman, 2020; Spruce & Bol, 2015). Methodological differences may contribute to this inconsistency. Self-reports are efficient and cover broader time frames, but are subject to recall bias, social desirability effects, and reference-point variability (Dignath & Veenman, 2020; Vandevelde et al., 2013). Observations, on the other hand, capture specific, overt behaviors in real time but may not reflect teachers’ intentions or practices across all contexts. Recognizing the limitations of observation studies, recent research advocates combining classroom observations with complementary methods such as teacher interviews (Heaysman & Kramarski, 2022). Such multi-method approaches enable researchers to capture both the enacted and intended aspects of SRL instruction, offering a more comprehensive picture of how teacher knowledge and practice align. Nonetheless, few studies (e.g., Spruce & Bol, 2015) have systematically applied this approach, particularly in primary education, leaving a significant gap in understanding how teachers’ SRL knowledge and beliefs translate into concrete classroom behaviors.

A key contribution of the present study is precisely this alignment analysis between teachers’ knowledge and beliefs and their observed practices, achieved by integrating video-based classroom observations with reflection-stimulated recall interviews. To our knowledge, only four previous studies have investigated the connection between observational data and teachers’ knowledge and beliefs, and with the exception of Spruce and Bol (2015), teachers’ knowledge was assessed exclusively through quantitative measures while beliefs were not examined qualitatively at all. By adopting a qualitative, multi-method approach, the present study provides a novel contribution, particularly within the underexplored context of primary education.

In doing so, this study responds to calls for research that simultaneously examines teacher competences (knowledge and beliefs), instructional contexts, and observed teaching behaviors (Dignath & Veenman, 2020), thereby providing a nuanced understanding of how SRL is, or is not, implemented in everyday classroom life. The following research questions are addressed in this study:

1. How and to what extent do primary school teachers implement SRL in their classroom practice?
2. What knowledge and beliefs about SRL do teachers hold, and how are these aligned with their observed classroom practice?
3. Which factors do teachers perceive as facilitating or constraining SRL implementation, and how can these explain variation in SRL promotion?

## 3. Materials and Methods

### 3.1. Participants and Procedure

Eight primary school teachers from four different schools participated in the study, seven of whom were female. The teachers were recruited from primary schools in Flanders (Belgium) through convenience sampling, as they were contacted on the basis of their prior participation in professional development initiatives. As these initiatives were implemented at the school level, individual teachers may not have personally participated in SRL-focused training. They had a mean age of 31.5 years, with an average of 9.75 years of overall teaching experience and 5.75 years of experience in their current grade. Teachers from Grades 1–6 were included to ensure representation across the full primary range and to capture the diversity of SRL-supportive practices throughout primary education. More specifically, the sample included two first-grade teachers, one third-grade teacher, three fourth-grade teachers, and two sixth-grade teachers.

After obtaining consent from the teachers, each was video-recorded during two lessons to investigate their implementation of SRL. Selecting two lessons per teacher aligns with previous observation-based research on SRL (e.g., Dignath & Büttner, 2018) and allowed us to balance sufficient depth of analysis with the practical feasibility of the study.

To ensure ethical compliance, the study protocol was reviewed and approved by the Ethics Committee of the Faculty of Psychology and Educational Sciences, Ghent University (protocol code 2023-054, 4 October 2023). Teachers were video-recorded from the back of the classroom using a high tripod, minimizing students’ presence in the video, and any potentially recognizable images of students were blurred prior to analysis. Prior to the observation, teachers were asked to deliver a lesson in which they implemented SRL-related strategies and activities as optimally as possible. This request was made because previous research (e.g., Dignath & Büttner, 2018) has shown that teachers rarely incorporate SRL strategies in their everyday practice. We aimed to explore the extent to which teachers could implement SRL strategies when explicitly encouraged to emphasize them in their teaching. However, several teachers later reported that they made few or no adaptations to their instruction compared to a typical everyday lesson.

All 16 lessons were video-recorded, resulting in 939 min of video material, with an average duration of 59 min per lesson. Teachers were free to choose which lesson to record, resulting in a broad range of subjects and instructional formats. The most frequently observed subject was mathematics, characterized by teacher-centered teaching and individual silent work by students.

An overview of the participating teachers, including background characteristics and descriptive information about each observed lesson, is provided in Table 1.

In addition to the video recordings, video-stimulated reflection interviews were conducted with each teacher within two weeks after the second observation. Immediately following each observed lesson, teachers were invited to indicate specific moments in the lesson they considered relevant to their implementation of SRL and that they would like to elaborate upon during the interview. After the second observation, teachers received a link to the recordings of their own lessons, allowing them to watch the videos again if they wished. The interviews lasted on average 1 h and 19 min and all interviews were audio-recorded and transcribed verbatim for analysis. This resulted in a total of 133 pages.

### 3.2. Instruments

#### 3.2.1. Observation Instrument

To analyze teachers’ direct and indirect SRL promotion, the instrument “Assessing How Teachers Enhance Self-regulated Learning” (ATES) was used (Dignath et al., 2022). The instrument focuses primarily on teacher behavior, teacher instructions, and instructional design. A distinction is made between indirect and direct SRL promotion.

##### Direct Instruction of SRL

The ATES instrument consists of a low-inference coding scheme for capturing direct instruction. It measures the quality and quantity of strategy instruction using fixed time intervals of 60 s. The instrument involves multiple coding dimensions, including the type of strategy promoted (i.e., cognitive, metacognitive, or motivational) and its corresponding subtype (Dignath et al., 2022). These types and subtypes are based on Boekaerts’ (1999) three-layer model of SRL. Additionally, the teacher’s strategy instruction is coded in terms of mode of instruction (i.e., explicit, implicit). We adapted the ATES instrument of Dignath et al. (2022) by excluding activation as a mode of instruction as well as the dimension of instructional approach (e.g., demonstrating, explaining). The instructional context is considered during the coding process, but it served only as background information and was not included in the subsequent analyses.

##### Indirect Instruction of SRL

In contrast to low-inference coding, which involves minimal subjective interpretation, high-inference coding requires coders to make more interpretative judgments in order to qualitatively evaluate certain observed classroom behaviors. High-inference coding was conducted at the end of each video to analyze teachers’ indirect promotion of SRL using a rating instrument consisting of six constructs and 21 items. Four of these constructs (i.e., self-determination, value, cooperative learning, and constructivist learning) were adopted from the ATES framework (Dignath et al., 2022). Building on this instrument and in line with research of Rosenthal et al. (2024), two additional constructs (i.e., success expectation and student support) were integrated to better capture the broader range of SRL-promoting practices. Each construct comprised three to five items, which were rated on a four-point Likert scale ranging from 1 (low) to 4 (high). An overview of the constructs and an example item per construct can be found in Appendix A.

#### 3.2.2. Teacher Interview

Semi-structured interviews with the participating teachers were conducted with three main objectives, each addressed through specific questions. First, the interviews aimed to explore teachers’ conceptual understanding of SRL. Based on Dignath-van Ewijk and van der Werf (2012) teachers were asked to describe in their own words what SRL means to them and what they believe it involves in their teaching. Second, the interviews aimed to stimulate teachers’ reflection on their SRL practices as captured in the video observations. The researchers selected three to five video fragments for each teacher, guided by Zimmerman’s (2002) cyclical model of SRL. Specifically, one fragment was selected from the beginning of the lesson to represent the forethought phase, one from the middle of the lesson to illustrate the performance phase, and one from the end of the lesson to capture the self-reflection phase, ensuring variation across key stages of the SRL process.

In addition to these theoretically selected fragments, one or two further fragments were chosen based on events observed during the lesson. These could reflect particularly strong examples of SRL-related instructional practice or missed opportunities to foster students’ SRL. Teachers were also invited to select and discuss fragments of their own choosing. The discussion of these fragments followed the principles of video-stimulated reflection interviews (Powell, 2005), allowing teachers to articulate their pedagogical reasoning and perceptions of SRL-related practices. More specific questions about intensions and purposes (e.g., What were your underlying intentions or objectives when applying this strategy or performing this action?) and questions prompting technical reflection (e.g., What was your rationale for selecting this strategy or addressing this topic in your lesson?) were posed in relation to selected video fragments. These fragments were chosen collaboratively by both the researchers and the teachers. Third, the interviews aimed to identify challenges and success stories related to promoting SRL in daily classroom practice. Teachers were encouraged to share concrete examples of what they found effective, difficult, or personally meaningful in fostering SRL among their students.

### 3.3. Data Analysis

#### 3.3.1. Video Observations

Teachers’ SRL promotion practices were analyzed with the ATES instrument (Dignath et al., 2022). All video-recorded lessons were coded independently by two trained Master’s thesis students. Prior to the main coding phase, coders underwent training using a coding manual and coded some videos together with the first author of this manuscript. Interrater reliability was calculated for a randomly selected subset of 25% of the observed lessons using Cohen’s Kappa to ensure consistency in both low- and high-inference ratings. Agreement scores indicated acceptable reliability across coding categories (κ = 0.78) (Landis & Koch, 1977). Any discrepancies were subsequently discussed until consensus was reached, and this agreed-upon coding scheme was then used for the remainder of the data. For the low-inference coding of direct SRL promotion, the frequency of observed strategy instruction was aggregated per observation and per teacher, consistent with prior research (Dignath et al., 2022). Descriptive statistics were computed for each strategy type (i.e., cognitive, metacognitive, motivational) and their corresponding subtypes, as well as for the mode of instruction (i.e., explicit or implicit). To ensure comparability across lessons of varying lengths, the coded data and associated frequencies were standardized to a 50 min school lesson. These standardized scores were then used to examine variations in SRL instruction both within and across classrooms.

For the high-inference coding, descriptive analyses were conducted to assess the extent to which teachers indirectly promoted SRL through their classroom environment. For each of the six constructs, a mean score was calculated across the corresponding items comprising the construct. This yielded a single composite score per construct, per lesson, reflecting the degree to which each SRL-supportive feature was present. Subsequently, these construct scores were averaged across the two observed lessons for each teacher to generate a teacher-level score for each dimension of indirect SRL promotion.

#### 3.3.2. Interviews

A coding scheme was applied to thematically analyze the interview data using MAXQDA. Thematic analysis was selected for its ability to identify recurring patterns while also accounting for the complexity and contextual nuances of teachers’ reflections on their implementation of SRL in classroom practice (Braun & Clarke, 2006). Both within-case and cross-case analyses were conducted (Miles & Huberman, 1994). Initially, individual interviews were synthesized into teacher-specific summaries to facilitate within-case analysis (Miles & Huberman, 1994). These summaries provided the basis for theme development aligned with the study’s research questions. The subsequent cross-case analysis was guided by a combination of deductive and inductive approaches. Deductively, a set of a priori codes was developed based on theoretical frameworks underlying the ATES instrument (Dignath et al., 2022), including Boekaerts’ (1999) three-layer model of SRL to code teachers’ conceptualizations of SRL, and literature on direct and indirect SRL promotion to code teachers’ reflections on their own instructional practices (e.g., Dignath et al., 2022; Karlen et al., 2023). Each broad category was further refined into subcategories through an iterative process of reading and re-reading the interview data. In parallel, inductive coding enabled the identification of novel themes that emerged directly from the data, thereby capturing context-specific insights beyond the predefined frameworks. An overview of the final coding categories, including their theoretical basis (deductive vs. inductive), is provided in Appendix B.

#### 3.3.3. Cross-Data Comparison

In a final step, the interview and observation data were integrated to enable a more comprehensive understanding of each teacher’s SRL implementation and to address the study’s research questions. First, within-case comparisons were conducted by juxtaposing the coded observational data with the corresponding teacher’s interview reflections in a teacher-specific summary. This facilitated the identification of consistencies, contradictions, and explanatory links between observed classroom practices and how teachers articulated their intentions, beliefs, knowledge, or challenges. Subsequently, cross-case patterns were examined to identify recurring themes and variations across teachers, drawing on insights from both data sources. This integrative approach supported a richer interpretation of the findings and strengthened the validity of the analysis through data triangulation.

## 4. Results

### 4.1. Research Question 1: How and to What Extent Do Primary School Teachers Implement SRL in Their Classroom Practice?

#### 4.1.1. Direct Instruction of SRL

Across the observations, no substantial differences were found between the first and the second lesson of teachers. Therefore, the average scores across both lessons were used for further analysis.

##### Type of Strategies Used

Overall, teachers varied in the types of SRL strategies they used. Some focused predominantly on cognitive strategies, others on metacognitive or motivational strategies, and a few showed a more balanced approach. For instance, Teachers E and G emphasized cognitive strategies, while Teachers A and B primarily focused on metacognitive strategies. Teachers C, D, F, and H tended to emphasize motivational strategies.

**Figure 1:**
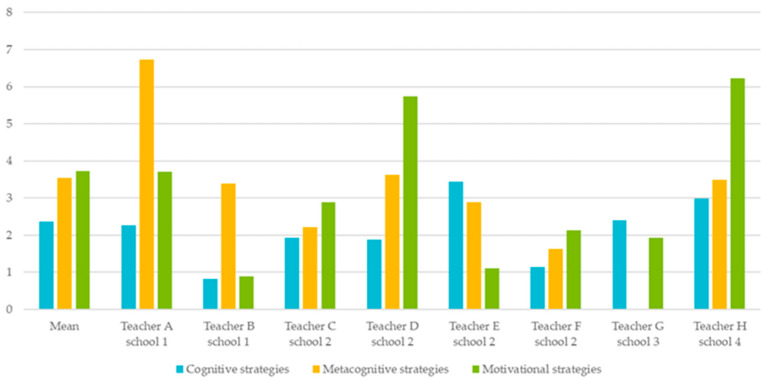
Direct instruction of SRL strategy types (average per teacher).

Figure 1 presents an overview of the average number of strategies in which teachers provided direct instruction, highlighting both overall patterns and individual variation. To examine the occurrence of direct instruction in cognitive, metacognitive, and motivational strategies, standardized scores were calculated. Each score reflects the average number of different SRL strategies for which a teacher provided direct instruction per lesson. On average, teachers offered direct instruction in 2.37 cognitive strategies, 3.54 metacognitive strategies, and 3.73 motivational strategies per lesson. While these averages highlight general trends, substantial variation existed across individual teachers.

For cognitive strategies, Teacher B provided the fewest instances of direct instruction (0.89 on average), whereas Teacher E offered the most (3.44). A common example of cognitive instruction was the use of repetition, such as referring back to content from previous lessons at the start of a new topic.

In terms of metacognitive strategies, Teacher A demonstrated the greatest variety of direct instruction, whereas Teacher G did not provide any metacognitive instruction in either lesson. For example, Teacher A asked students to complete both daily and weekly planning sheets and frequently referred back to these throughout the lesson to guide learning.

Regarding motivational strategies, Teacher H provided direct instruction across the broadest range of strategies (6.23), while Teacher B again offered the fewest instances (0.89). Teacher H’s approach included extensive use of social resources, prompting students to consider who they could ask for help if they did not understand a task, and referring to classroom tools or support systems where students could seek assistance.

No clear relationship emerged between the type of strategies teachers implemented and either the grade level taught or their years of teaching experience. Interestingly, some patterns of strategy emphasis appeared to cluster within schools. For example, Teachers A and B, who focused on metacognitive strategies, and Teachers C, D, and F, who emphasized motivational strategies, each belonged to the same school. While this may suggest that school context influences SRL implementation, this interpretation is based on descriptive patterns rather than formal statistical testing and should be considered exploratory.

##### Mode of Instruction

**Figure 2:**
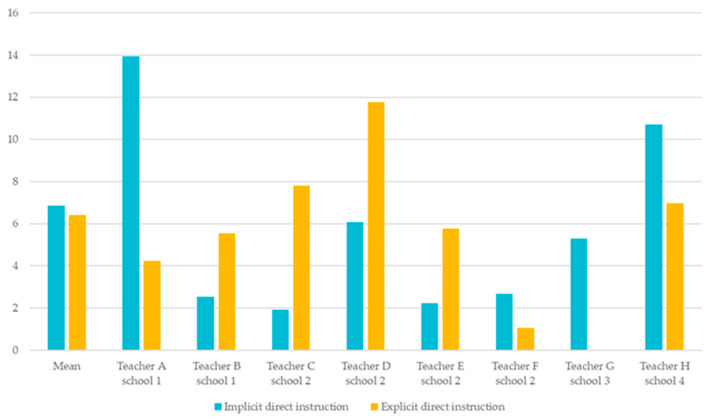
Modes of instruction (average per teacher).

On average, instructions occurred 6.84 times implicitly and 6.42 times explicitly, indicating a roughly equal distribution. Figure 2 presents an overview of the average number of strategies taught explicitly and implicitly, based on the coding of each strategy type. Considerable variation was observed at the individual level. Teachers A, F, G, and H tended to instruct SRL strategies primarily in an implicit manner, whereas Teachers B, C, D, and E adopted a more explicit approach.

An example of implicit direct instruction was observed for Teacher A, who modeled self-monitoring by describing the steps she herself took when checking her work. For instance, she prompted a student with “And now? What do you want to do next?” and demonstrated how she consulted her study plan to check off the completed task. Through this modeling, students were guided to monitor their own learning while observing the teacher’s approach.

In contrast, Teacher E demonstrated explicit direct instruction during a mathematics lesson by clearly explaining not only how to calculate mean scores, but also why this skill is important and in which contexts it should be applied. This approach involved step-by-step guidance, ensuring that students understood both the procedure and its practical relevance.

Finally, no systematic relationship was found between these approaches and grade level, teaching experience, or school context, as both implicit and explicit strategies were present across teachers from different schools.

#### 4.1.2. Indirect Instruction of SRL

Table 2 shows the average levels of individual teachers on six dimensions of indirect instruction: cooperative learning, constructivist learning, self-determination, task value, success expectation, and student support. On average, teachers placed the greatest emphasis on student support (M = 3.39) and on making success expectations explicit (M = 2.77). In classroom practice, this often involved responding to students’ mistakes and turning them into learning opportunities, thereby scaffolding students’ understanding and progress. Making success expectations explicit was observed, for example, when teachers provided students with a variety of resources and tools, such as cheat sheets, calculators, or summaries, and offered targeted guidance to clarify what was expected of them.

In contrast, cooperative learning (M = 1.81) and highlighting task value (M = 1.81) were observed least frequently. Cooperative learning was rarely implemented in a way that actively engaged students in collaborative problem-solving or used multiple forms of peer interaction. Task value was infrequently addressed, with few teachers linking content to real-life contexts or providing learning materials from students’ daily experiences.

#### 4.1.3. Summary

As an overarching conclusion for research question 1, the findings indicate that teachers implemented SRL strategies to varying degrees and in diverse ways, reflecting clear preferences for particular strategy types and considerable variation between individual teachers. While no systematic patterns were found in relation to grade level or teaching experience, some trends appeared to cluster within schools. These observations suggest that school context may influence instructional practices. Both explicit and implicit modes of instruction were employed with roughly equal frequency, and student support consistently received high emphasis across teachers.

### 4.2. Research Question 2: What Knowledge and Beliefs About SRL Do Teachers Hold, and How Are These Aligned with Their Observed Classroom Practice?

#### 4.2.1. Teacher Knowledge

##### CK About SRL

The interviews indicated that all teachers demonstrated awareness and knowledge of the metacognitive component of SRL, consistently addressing the three central elements of planning, monitoring, and evaluation. Teacher E, for example, referred to SRL as:

*“On the whiteboard, there was an overview of the exercises they had to complete, and the students could choose for themselves which ones to start with. They also used the blocks on their desks to signal to each other whether they had understood something or not, which allowed them to adjust accordingly. In this way, they were learning to assess themselves: ‘Did I really get it?’ In addition, when they had to work through the two types of exercises, they could again reflect: ‘Do I really understand it now or not?’ That helped them monitor their own learning. The group discussions were also valuable, because they could compare answers: ‘You have this answer, I have that one, how did we actually get there?’ This kind of collective reflection after the exercise was important for them.”*

(Teacher E)

In contrast, cognitive aspects of SRL were explicitly mentioned by only a few teachers (Teachers C, E and G). Motivational knowledge was acknowledged by all teachers, though with notable differences in scope and emphasis. While most teachers referred to task value, Teachers A and B made no explicit reference to this element. Mentions of social resources, self-worth, and elements of the learning environment or volition/effort control occurred only sporadically.

When linked to classroom practice, partial alignment became apparent. Teachers such as A, D, and H showed the strongest consistency between their stated views in the interview and their observed practices: Teacher A emphasized metacognitive and motivational knowledge in the interview and also demonstrated the most implementation of metacognitive strategies in the classroom. Teacher D frequently referred to motivational strategies and likewise showed a strong emphasis on these strategies in practice, alongside moderate instruction in cognitive and metacognitive strategies. Similarly, Teacher H demonstrated the highest emphasis on motivational strategies in classroom practice, which aligned with multiple references to this component as central to SRL in the interview.

*“An important aim of instruction is to ensure that students can generate their own motivation through the way they think. This encompasses a wide range of practices aimed at enabling students, once they leave the school context, to self-regulate effectively, with appropriate motivation, clear goals, and a well-structured plan for organizing and approaching tasks, so that they internalize these skills and can apply them independently.”*

(Teacher H)

Only partial alignment between the interview and observation data was found for teachers B, C, and E. Teacher B reported metacognitive and motivational knowledge but, respectively, demonstrated only moderate and low references to metacognitive and motivational strategies in practice. Teacher C emphasized all three components in the interview, yet in their practice prioritized motivational strategies while instruction in cognitive and metacognitive strategies remained low. Teacher E, similarly, focused on all components in the interview but showed the highest reference to cognitive strategies in practice and low implementation of instruction in metacognitive and motivational strategies. Finally, Teachers F and G exhibited low alignment: Teacher F made frequent references to metacognitive and motivational strategies in the interview but applied very limited instruction in strategies of any kind in practice. Teacher G reported knowledge across all three components, but failed to implement metacognitive strategies, showed limited instruction in motivational strategies, and demonstrated only moderate instruction in cognitive strategies. Table 3 presents an overview of teachers’ reported knowledge (type of strategy), their observed strategy use, and the extent to which these align.

##### PCK About SRL: Direct Instruction

The interview data indicate that half of the teachers (C, E, F, G) emphasized explicit direct instruction, whereas six of the eight teachers (A, B, C, E, F, H) highlighted implicit forms. Despite these reported preferences, teachers’ PCK regarding the mode of direct instruction shows varying degrees of correspondence with their observed classroom practice. Teachers A, C, E, and H demonstrated high alignment: teachers A and H, who stressed implicit instruction in their interview, consistently implemented this approach in the classroom, while teachers C and E, who emphasized explicit instruction, were observed applying explicit strategies accordingly. Alignment is lower for teachers B, D, F, and G. Teacher B, for example, referred to an implicit mode of SRL instruction, but was observed using explicit methods, whereas teacher D made reference to implicit direct instruction yet demonstrated explicit strategies in practice. Similarly, Teachers F and G reported explicit approaches but predominantly implemented implicit direct instruction during the observed lessons. A summary of the teachers’ reported and observed modes of direct instruction, along with the alignment between both, is provided in Table 4.

##### PCK About SRL: Indirect Instruction

Analysis of teachers referring to indirect instructional practices in the interview showed that self-determination is consistently the most emphasized dimension, with teachers frequently highlighting decision-making autonomy, open forms of teaching (e.g., weekly planning), offering choices in learning activities, and fostering student self-evaluation. Except for Teachers C and G, all teachers referred to cooperative learning, while constructivist approaches were referenced only by Teachers C and E. Other dimensions, including task value, success expectation, and student support, received more variable attention in the interviews. Student support was seldom explicitly mentioned but was sometimes linked to reframing mistakes as learning opportunities, for example, by Teacher E.

*“A key element is the self-directed aspect: how am I going to arrive at my solution, how to adjust my approach, and also the learning strategy itself: how did I apply it, how did you apply it, how should it have been applied, and what have I forgotten? As I mentioned earlier, students recognize moments like, ‘Ah yes, I forgot to add this small part.’ They become aware that, ‘I missed this connection in this task, but I did remember it in the next one.’ In other words, they understand what they needed to do but simply forgot. The focus here is on learning from mistakes; I constantly emphasize this. Making mistakes is allowed, but students must understand what the mistake was and why it occurred. They genuinely internalize this process here.”*

(Teacher E)

The balance between teacher guidance and student autonomy was seldom discussed, appearing only in the interviews with teachers B, D, and G. Some teachers emphasized continued teacher control while others advocated for greater student independence.

*“It is essentially a form of freedom or autonomy that you give to the students, but you cannot provide freedom without guidance. There must always be direction from the teacher; otherwise, the students will just do whatever they want.”*

(Teacher B)

Comparison of the interview data with the classroom observations revealed clear connections and some discrepancies. Self-determination appeared relatively consistent across teachers, reflecting its prominence in both reported knowledge and observed practice. Student support, although rarely explicitly mentioned in the interviews, emerged as one of the most observed practices in the classroom, suggesting that teachers engaged in supportive behaviors in the classroom without explicitly linking them to SRL. Conversely, cooperative learning and task value, which were highlighted by some teachers in interviews, were less frequently observed in classroom practice, indicating gaps between reported emphasis and actual classroom implementation.

At the individual level, the alignment between teachers’ reported knowledge of indirect instruction and their observed practices was limited. Teacher B demonstrated partial alignment, as self-determination was the most frequently mentioned dimension in the interview and also emerged most prominently in the classroom observations. However, this teacher could not be considered as showing strong alignment, since the second most frequently referenced dimension (i.e., cooperative learning) was addressed least in observed practice. For the other teachers, the most frequently observed dimensions—particularly student support as the most addressed and cooperative learning or value as the least addressed in practice—did not correspond to what was highlighted in interviews. Overall, the findings suggested that while teachers often articulated self-determination and success expectation as central to their indirect instruction, in practice their instructional emphasis was more strongly oriented toward student support. An overview of these findings is presented in Table 5.

##### Misconceptions

In addition to the relatively limited content and pedagogical content knowledge demonstrated by the teachers, several misconceptions about SRL emerged from the interviews. The most common misunderstanding, expressed by nearly all teachers except F and H, was the tendency to equate SRL with student independence. Teacher G, for instance, framed independence as the very essence of SRL:

*“Learning to learn, but that is of course very broad. Yet it means that they also learn to be independent. Not having to ask all the time ‘what should I do now,’ but really learning that when something is written on the board, they should look at it themselves. So, that they truly learn independent.”*

A second misconception concerned the centrality of student well-being. Four teachers (B, C, D, and H) explicitly positioned well-being as the core of SRL, with Teacher D stressing that:

*“For me, the most important thing about SRL is the well-being of children. That they truly feel that they matter.”*

A third misconception related to the role of rules and classroom routines in fostering SRL. Some teachers, such as Teacher H, associated SRL with reducing traditional routines, for example, by no longer requiring students to line up after recess but instead allowing them to return to class independently and calmly.

*“When the bell rings after recess, the children are allowed to walk downstairs calmly. They do not need to line up anymore. They just go quietly to their own classroom. In this way, they experience that it can also be done in a calm and pleasant manner, and that lining up in a strict way is not really necessary.”*

(Teacher H)

Others, however, such as Teachers B, E, and G, argued that SRL depends on providing clear and consistent routines, describing practices like organizing a goals board or buddies board as essential scaffolds for student regulation. In several cases, teachers also conflated SRL with general classroom management practices, such as greeting the teacher or walking in an orderly manner, thereby blurring the distinction between everyday routines and instructional strategies that genuinely promote SRL.

#### 4.2.2. Teacher Beliefs

##### Positive Beliefs About SRL

Four of the eight teachers explicitly expressed positive beliefs toward the implementation of SRL throughout their interviews. They emphasized the benefits of SRL, describing it as an approach that increases student motivation, enhances learning outcomes, and supports all learners. As Teacher D stated:

*“I have been observing this for three years now. The more I start working in this SRL way, the further ahead my students are compared to other classes, and the better their results become. So yes, I do notice the advantage.”*

The remaining teachers did not explicitly express such positive beliefs about SRL, but neither did they express clear negative beliefs. Despite the positive statements, these positive attitudes were not reflected in classroom practice: teachers who emphasized the advantages of SRL did not demonstrate higher levels of direct or indirect instruction in their observed classroom practice.

##### Self-Efficacy Beliefs to Implement SRL

A recurring theme in the interviews concerned the uncertainties teachers experience about their ability to implement SRL effectively. All teachers except for teacher B reported doubts about their approach, with teachers C, D, and E expressing the strongest concerns. Teacher D, for instance, was particularly unsure about how to guide students in setting goals:

*“I am still searching for how to approach this. It is also expected that SRL should be made visible. For example, you need to set goals, but you also have to make them visible to the students. So, I do not yet know how to do that.”*

Similarly to teacher D, teachers C and E expressed uncertainties about their overall instructional approach to implementing SRL and how they could put it into practice. Notably, these three teachers all belonged to the same school and showed comparable patterns in classroom practice: they relied more heavily on explicit than implicit direct instruction and provided strong student support within indirect instruction. Taken together, these findings suggest that limited self-efficacy beliefs shaped teachers’ enactment of SRL, potentially constraining the extent to which their positive orientations translated into concrete practices.

#### 4.2.3. Summary

In summary, as an answer to research question 2, teachers’ knowledge and beliefs about SRL appeared to be only partially aligned with their classroom practice. While metacognitive and motivational components were widely recognized, cognitive strategies received less consistent attention. Alignment varied across teachers, with some showing strong consistency and others demonstrating low correspondence, particularly for metacognitive and motivational strategies. Implicit and explicit modes of instruction and indirect instruction such as focusing on self-determination of students and providing student support were enacted differently than reported. Overall, teachers exhibited more misconceptions—such as equating SRL with independence or routines—and limited self-efficacy than explicit positive beliefs.

### 4.3. Research Question 3: Which Factors Do Teachers Perceive as Facilitating or Constraining SRL Implementation, and How Can These Explain Variation in SRL Promotion?

#### 4.3.1. Factors on Student Level

##### Student Age

Although several teachers work with first-grade students, only one, Teacher A, mentioned in the interview that the age of her students can sometimes influence the implementation of SRL. She explained that, due to the young age of her learners, tasks must be introduced in very small, incremental steps. For example, at the beginning of the school year, many students are not yet able to read, which can make following a structured planning board challenging. In the observational data, Teacher A still demonstrated frequent use of metacognitive strategies during her lessons, suggesting that age-related challenges do not necessarily preclude the implementation of SRL strategies.

##### Student Ability Level

Five teachers (A, B, C, D, and E) identified the overall ability level of their students as a potential barrier to implementing SRL in classroom practice. They reported that when focusing on SRL, weaker students often make limited progress. As Teacher A explains:

*“I often try to get up from my seat and check on everyone to see if they are managing, but when I let go of the weaker students, they often do not engage with the tasks. By the end of the day, they have not completed their assignments, which is very challenging. The gap between the very weak students and the rest of the class is large. As a teacher, this is extremely difficult. People sometimes say you should also let the weaker students work independently, but then they produce nothing, and that is hard for me. I want them to learn and to make progress in their development.”*

This reasoning illustrates a common misconception: namely, that students’ ability levels inherently limit the extent to which they can benefit from SRL. Underlying this view is the related misconception that SRL primarily equates to independent work. When SRL is understood in this narrow sense, weaker students appear to be excluded from its potential benefits.

The perceived challenge is not limited to general ability levels; differences in students’ existing SRL skills also contribute to difficulty in implementation. Teacher C, for example, noted that large variations in students’ SRL competence make targeted support more challenging. In addition, Teacher D raises concerns regarding students with learning disorders:

*“As I work more with SRL, I increasingly notice that children with learning disorders, such as autism or dyspraxia, tend to struggle. I wonder if research has examined whether these students can navigate SRL effectively, because I get the impression that they may actually regress rather than progress.”*

In trying to explain variation in teachers’ SRL implementation, the interview data suggest that several teachers perceive the academic level of their students as a barrier. However, the observation data indicate that these perceptions do not systematically constrain actual classroom practice. Despite their concerns, teachers continue to employ a range of cognitive, metacognitive, and motivational strategies, suggesting that student-level characteristics function more as a perceived than an actual barrier to SRL enactment.

##### Home Environment/Parents

Finally, five teachers (A, B, C, E, and G) referred to the home environment, and more specifically to the role of parents. They noted that when parents frequently take over tasks from their children, both at home and, for example, upon arrival at school (e.g., preparing the correct materials), students are hindered in further developing the SRL skills practiced in class. Teacher C explains:

*“I think they sometimes do not realize what their children are actually capable of. They prefer to do things themselves rather than teaching their children to manage on their own. Because it is faster, easier, and often neater if they do it themselves. I think this reflects a broader societal tendency: wanting to step in for children and prevent them from facing difficulties. But also at home, when something goes wrong, parents immediately step in, immediately provide explanations and reactions. As a result, I believe children become less independent than they could be.”*

However, similar to student age and ability, no clear connections are observed between teachers’ references to the home environment and their actual SRL implementation in classroom practice. Even though teachers perceived that parents may hinder students in further developing the SRL skills practiced in class, this perception was not reflected in our observations, where no systematic differences in the use of SRL strategies could be identified.

#### 4.3.2. Factors on Teacher/Classroom Level

In addition to the previously discussed teacher-level factors, such as their knowledge and beliefs, the teachers repeatedly referred to other teacher- or classroom-level factors that, in their view, influence the extent to which they implement SRL in their classroom practice.”

##### Classroom Space and Organization

When teachers were asked about the ideal implementation of SRL in their classroom practice, or about what they would need in order to promote SRL more effectively, four teachers (C, D, E, and H) mentioned the need for a larger classroom, viewing their current physical setting as a potential barrier. More specifically, Teacher C explained:

*“I would like a larger classroom, perhaps with a partition wall, so that students could choose where to sit based on their needs: those who want to follow the whole-class instruction sit there, others join me to move ahead, and those ready to go further can sit elsewhere. Students would need to assess themselves to make that choice. This setup would allow me to distribute explicit instruction across the two sections, which would really support students’ SRL. That would definitely be my preference.”*

This response reflects a possible misconception, namely that a larger classroom is a prerequisite for implementing SRL more effectively. Notably, three of the four teachers who emphasized this issue all teach in the same school.

##### Use of Textbooks and Teaching Methods

Two teachers (Teachers D and E) also identified the reliance on textbooks and prescribed teaching methods as a barrier to fostering SRL, arguing that it limited their ability to respond to students’ task interests. Teacher D described this challenge as follows:

*“The second issue I encounter is the use of methods. The children’s task interest decreases when they simply have to complete the workbook, instead of being asked what they want to learn. I think those are the main challenges.”*

Given that teachers within the same school typically use the same methods and textbooks, it is unsurprising that both of these teachers came from the same school. However, it is notable that the other two teachers from this school did not report this as a barrier, suggesting that perceptions of this limitation may be influenced by individual teaching styles or classroom approaches rather than the school context.

##### Workload and Time Investment

Finally, five of the eight teachers perceived the implementation of SRL as an additional workload, requiring considerable time investment. Preparing student planning documents, finding ways to assess students’ SRL skills, and checking students’ self-evaluations were all experienced as demanding tasks. Teacher A noted:

*“Of course, it is extra work and you have to prepare the planning. In other schools, this is not the case. In our school, we need to prepare the weekly planning in the evenings and at weekends. This takes a lot of time and extra work. You also have to plan in advance, like ‘that exercise I will give to the students in track 1,’ or ‘that exercise is too difficult for the students of track 1, so I will leave it out.’ You spend more time preparing than just writing in your agenda what lesson you will teach. It is definitely more preparatory work, but in the end the effort is rewarded because the students can actually work with it.”*

These experiences could not be linked to school context, teaching experience, or teacher age, nor to whether and how teachers implemented SRL in their classrooms.

#### 4.3.3. Factors on School Level

##### School Leadership

Two teachers (D and G) identified support from the school leader as a key facilitator for further implementing SRL in their classrooms. They emphasize, first, the need for direct support in developing SRL practices, and second, the role of the school leader in providing professional development opportunities, embedding SRL within the school’s vision and ensuring continuity in the teaching approaches.

##### Vision of the School

Three teachers (F, G, and H) explicitly highlighted the importance of embedding SRL in the school’s vision and policy. Teacher G emphasized this point:

*“That the school makes clear agreements about it. That it is included in the school policy. Which is ultimately the intention. But that it is considered just as important as mathematics, for example, and that it might even be included in student reports. Not necessarily by giving grades, but by highlighting it as an important area of attention to parents.”*

##### Continuity Through Learning Trajectories

While school leadership and vision are rarely mentioned in the interviews, all teachers unanimously agree on the importance of developing a coherent learning trajectory across grades to support SRL implementation. They emphasized that continuity across classes is crucial. For instance, Teacher A and Teacher B described using consistent practices, such as traffic lights for signaling questions or assigning “helping peers,” because these strategies are coordinated across grades.

In contrast, teachers from School 2 (Teachers C, D, E, and F) noted that such continuity is currently lacking, which they experienced as burdensome, although efforts are being taken to improve it. Teacher F illustrates this concern:

*“Establishing a coherent learning trajectory across the school is a long-term process. I hope that my colleagues in fourth grade will be able to benefit from it and build on it, and that I can also build on the work done with the children I will teach next year. This way, they won’t feel like everything is completely new. I think that will already make a big difference. Right now, that common thread is still missing.”*

##### Collaboration and Support

To develop continuous learning trajectories across grades and to ensure continuity, seven teachers referred to the importance of collaboration and support at the school level. Teacher E, for example, describes:

*“Yes, we have an SRL group, actually a working group. Every grade is represented in it. And we have meetings every month, or every two months, to discuss the actions we have implemented around SRL. Then we evaluate and reflect on how we can adjust them so that it really becomes one coherent approach.”*

Collaboration was perceived, on the one hand, at the team level, for instance through the exchange of SRL practices. On the other hand, several teachers indicated that support from another teacher in the classroom itself is also appreciated, as this would help them to further strengthen their focus on SRL implementation.

##### Explaining Variation in SRL Promotion

Overall, school-level factors did not provide a clear explanation for the observed variation in teachers’ promotion of SRL across classrooms, even though nearly all teachers acknowledged their importance. However, one noteworthy observation emerges from Teacher G’s responses: in cases where collaboration with colleagues around SRL was limited, the lowest levels of direct instruction were reported for cognitive, metacognitive, and motivational strategies.

#### 4.3.4. Summary

In summary, regarding research question 3, teachers reported multiple factors at the student, classroom, and school levels as facilitating or constraining SRL implementation. At the student level, age, ability, prior SRL skills, and parental involvement were perceived as potential barriers, although observational data suggested that these factors did not systematically limit teachers’ use of SRL strategies. At the classroom level, constraints included physical space, reliance on textbooks or prescribed methods, and the additional workload required to plan and monitor SRL activities. At the school level, leadership support, a clear vision for SRL, continuity across grades, and collaboration among teachers were highlighted as important facilitators, yet these factors alone did not appear to fully explain variation in SRL promotion. Overall, the findings suggest that while teachers perceive numerous potential barriers, actual classroom practice is shaped by a combination of individual, contextual, and institutional factors.

## 5. Discussion

The following discussion elaborates on bringing together insights from both the interviews and video-based classroom observations to examine teachers’ knowledge, beliefs, and practices regarding SRL in primary education. In doing so, we discuss themes that emerge across the research questions. We also reflect on the study’s limitations, identify avenues for future research, and outline practical implications for supporting opportunities for sustainable SRL promotion in practice.

### 5.1. Variation in SRL Implementation

In line with the review study by Dignath and Veenman (2020), which synthesizes findings from multiple observation studies, teachers in our study rarely engaged in direct instruction of SRL strategies. When direct instruction did occur, the emphasis appeared to be predominantly on metacognitive and motivational strategies, with cognitive strategies receiving less attention. Interestingly, our findings revealed a somewhat stronger focus on motivational aspects compared to Rosenthal et al. (2024), who reported metacognitive strategies as most prominent. Considerable variation was observed across teachers, which may be linked to differences in school context. This suggests that school-level factors may act as important levers for promoting particular dimensions of SRL, although empirical research in this area remains limited. Direct SRL instruction was observed in both explicit and implicit forms, yet teachers differed markedly in their preferences and the consistency. This finding aligns with previous research demonstrating that an optimal balance between explicit and implicit instruction maximizes student learning outcomes (Kistner et al., 2010). However, such balanced instruction was rare in our sample, underscoring the need for professional development that strengthens teachers’ ability to employ all three components of direct SRL instruction—cognitive, metacognitive, and motivational—while also diversifying their use of explicit and implicit methods. Indirect approaches to fostering SRL, classroom practices were largely oriented toward general student support, whereas cooperative learning and fostering task value were notably underrepresented. This pattern mirrors findings by Rosenthal et al. (2024), who likewise reported that student support was most frequently addressed, while comparatively little attention was given to emphasizing the value of learning content. These patterns may indicate systematic blind spots in teachers’ enactment of SRL potentially limiting students’ development across self-regulatory domains. Given that task value and opportunities for collaboration are critical drivers of motivation and engagement (Eccles & Wigfield, 2020), their absence may limit students’ ability to develop robust and transferable SRL skills.

### 5.2. The Role of the School Context

While individual variation in SRL implementation was prominent, teachers from the same school tended to emphasize similar SRL strategies, suggesting that school-level factors such as school culture, leadership, and policy may play a role in shaping SRL practices. These influences also shaped teachers’ perceptions of key facilitators and barriers, with leadership support, cross-grade continuity, and collegial collaboration frequently identified as essential conditions for fostering SRL. Our findings resonate with research from De Smul et al. (2020) and Thomas et al. (2022), which showed that schools with explicit school policies, shared vision, and sustained collaboration among colleagues were more consistent in their SRL practices, with school leadership playing a particularly supportive role.

Although leadership practices were not directly observed in this study, the conditions described by participating teachers echo key features of two influential and enduring leadership models in educational research: instructional leadership and transformational leadership (Bush, 2014; Hallinger, 2003). Instructional leadership emphasizes guiding teaching and learning by setting clear pedagogical goals, aligning instructional practices, and supporting teachers in achieving those goals (Hallinger & Heck, 2011), for example, by establishing explicit expectations regarding SRL, ensuring coherence across grades, and enabling teachers to work collectively toward common SRL objectives. Transformational leadership focuses on inspiring and empowering staff by articulating a shared vision, nurturing a culture of trust and professional agency, and encouraging reflective inquiry and innovation (Hallinger & Heck, 2011), for instance, by fostering a strong and shared commitment to SRL, facilitating collaborative learning and dialogue among teachers, and creating a climate in which teachers feel motivated to continuously improve their SRL practices. Promoting SRL therefore cannot be reduced to strengthening individual teacher practice alone; it may require a coordinated, school-wide approach in which leadership commitment, cross-grade continuity, and collective professional learning function as key levers. Further research is needed to examine how specific leadership practices shape SRL implementation in practice, and how instructional and transformational leadership may interact in supporting school-wide SRL development.

### 5.3. Connecting What Teachers Think and What Teachers Do

Teachers’ knowledge of SRL in our study appeared to focus on metacognitive and motivational strategies, while cognitive strategies received less attention. This contrasts with previous research indicating that teachers’ weakest knowledge often lies in the metacognitive component (Barr & Askell-Williams, 2019; Dignath & Büttner, 2018). Teachers mentioned both implicit and explicit forms of direct instruction, but rarely referred to indirect dimensions such as fostering task value or promoting cooperative learning. This raises the question of whether teachers might be aware of these dimensions but do not explicitly conceptualize or label them as part of SRL.

With respect to misconceptions, the most prevalent one found in our sample was the tendency to equate SRL with independent learning, a pattern also consistently reported in earlier studies (Callan & Shim, 2019; Dignath & Sprenger, 2020; Šimić Šašić et al., 2023; Spruce & Bol, 2015). At the same time, our study revealed additional misconceptions that are less frequently addressed in the literature, namely the idea that SRL primarily concerns supporting students’ well-being, that the physical classroom space is a determining factor, and that SRL depends on maintaining clear and consistent routines. Such misconceptions risk narrowing the scope of SRL and may prevent teachers from fostering students’ SRL skills in a systematic way. Addressing these misconceptions in professional development may help bridge gaps between knowledge and practice.

Teachers’ beliefs varied widely: while some teachers highlighted benefits of SRL, others expressed ambivalence or uncertainty. Only half of the teachers explicitly voiced positive beliefs, contrasting with prior questionnaire-based studies that generally report more strong teacher endorsement towards SRL implementation (Dignath-van Ewijk & van der Werf, 2012; Vosniadou et al., 2020). This discrepancy suggests that open-ended interviews may reveal more nuanced or cautious positions. Another key finding concerns teachers’ self-efficacy beliefs. A particular strength of this study is that we explored these beliefs qualitatively, whereas most prior research has relied primarily on quantitative self-report approaches. Nonetheless, our findings converge with earlier studies: many teachers expressed doubts about their ability to implement SRL effectively in practice, consistent with findings from De Smul et al. (2018). These doubts were often clustered within schools, suggesting that self-efficacy beliefs are shaped not only individually but also collectively through school-level dynamics.

Finally, a key contribution of this study lies in its analysis of the alignment between teachers’ reported knowledge and beliefs and their observed practices, achieved by combining classroom observations with reflection-stimulated recall interviews. To our knowledge, only a few previous studies have explicitly investigated this alignment, making our approach particularly novel. Building on Spruce and Bol (2015), our findings show discrepancies between what teachers articulated and what they enacted: some teachers reported broad SRL knowledge but did not consistently demonstrate it in practice, whereas others effectively implemented SRL strategies without explicitly labeling them as such in the interviews. This misalignment, also reflected in prior research (e.g., Karlen et al., 2020; Dignath-van Ewijk, 2016), highlights that strengthening sustainable SRL implementation requires not only knowledge but also systematic reflection and coherence across beliefs and practices.

### 5.4. Barriers to Implement SRL

The observed misalignments, limited knowledge, and school-level factors, as discussed earlier, may constitute important barriers. At the student level, teachers in our study pointed to age, ability, parental involvement, and learning difficulties as potential obstacles. Interestingly, these concerns did not always align with classroom practices, suggesting that some barriers may be more perceived than real. This finding resonates with (Vandevelde et al., 2012), who emphasized the diversity between students as one of the main barriers to implementing SRL, alongside perceived time pressure. Such perceptions underline the need to support teachers in critically reflecting on whether student characteristics genuinely restrict SRL, or whether assumptions may unnecessarily limit its implementation.

At the teacher and classroom level, a range of barriers was reported. Several teachers highlighted the considerable workload and time investment required for SRL, such as preparing planning documents, which mirrors the time-related barriers consistently found in the literature (Del Mario & Tran, 2024; Vandevelde et al., 2012). In addition, some teachers considered classroom space and organization as hindering factors. This reflects a possible misconception, as a larger classroom is not a prerequisite for effective SRL implementation. Other reported barriers in our study included the reliance on textbooks and prescribed teaching methods. Moreover, because barriers were often framed collectively within schools, addressing them requires whole-school strategies. Sustainable SRL implementation likely depends not only on individual teachers but also on the active engagement of students, parents, and leadership.

### 5.5. Practical Implications of the Study

These findings have several practical implications. First, professional development should be multifaceted, targeting not only teachers’ knowledge of SRL, including underrepresented aspects such as cognitive strategies, task value, and cooperative learning, but also addressing common misconceptions, such as equating SRL with student independence or reduced structure. Based on our findings, such professional development should include concrete examples and modelling of SRL instruction, particularly in cognitive domains, and offer practical formats such as video-based peer feedback and reflection sessions with other teachers. Given the considerable variation among individual teachers, professional development should also help them diversify their instructional approaches. This includes combining explicit and implicit direct SRL instruction and integrating motivational elements like choice and relevance into lesson design.

Second, given the observed limited alignment between teachers’ knowledge, beliefs, and actual classroom practices, professional learning should incorporate reflective activities to help teachers critically evaluate and align these aspects. These may include reflection-stimulated recall interviews, SRL observation checklists, and peer consultation formats that help teachers critically evaluate and align their conceptual understanding of SRL with enacted practice. Such tools can also help uncover implicit SRL enactment and foster more intentional implementation.

Third, enhancing teachers’ self-efficacy is essential, which can be achieved through concrete, practice-oriented tools, modelling, and collaborative learning opportunities that build confidence in implementing SRL (Bandura, 1977). At the same time, sustainable SRL implementation is unlikely to be achieved through isolated teacher-level initiatives alone. A school-wide approach, with a shared vision, clear goals, strong leadership support, and structured opportunities for collaboration across grade levels, is likely to be more effective in fostering lasting practices. Our findings highlight the importance of leadership commitment and cross-grade continuity, which can be operationalized through policy documents, team agreements, and time allocation for joint planning and reflection.

Finally, achieving meaningful SRL development also involves engaging students and parents. Schools can support this by introducing SRL goal-setting tools for students, organizing parent information sessions on SRL, and providing home-based strategies to reinforce SRL skills. In doing so, responsibility and support for SRL become shared across the school community, enhancing the sustainability and impact of SRL practices.

### 5.6. Limitations and Suggestions for Future Research

While this study provides valuable insights into teachers’ knowledge, beliefs, and practices regarding SRL, several limitations should be noted.

First, the small and uneven sample limits the generalizability of the findings. Data were collected from only eight teachers across four schools in Flanders, Belgium. The schools had previously participated in SRL-related professional development initiatives. Although most teachers reported little or no prior experience with SRL, the selection of schools may have influenced the results. Future research could include a larger and more diverse sample, encompassing schools that have not participated in professional development projects, as well as schools in other regions or countries.

While this study included teachers from across the entire primary range (Grades 1–6) to capture a broad perspective on SRL-related teaching practices, we acknowledge that students’ SRL development is strongly influenced by age (Perry et al., 2017). As such, the inclusion of all primary grades may have masked some grade-specific nuances in SRL implementation. Future research could therefore focus on more specific grade levels or narrower age bands to better understand how SRL-supportive practices evolve as students progress through primary school. Such research could also examine whether and how student age or grade level is experienced as a challenge for SRL implementation, thereby offering a more nuanced understanding of the interplay between learner characteristics and teachers’ instructional practices.

In addition, classroom observations encompassed only two lessons per teacher, which likely did not capture the full range of variation in teachers’ SRL implementation. It should also be noted that the observed lessons were not examples of teachers’ regular classroom instruction, but rather of lessons that teachers had been asked to design and deliver with an explicit focus on SRL-related strategies. Although some participants indicated that they made few or no adaptations, these observations likely reflect teachers’ capacity to implement SRL when explicitly prompted, rather than their spontaneous, everyday practices. However, since teachers were asked to demonstrate their best SRL practices, it can reasonably be assumed that the observations provided a representative picture of their instructional repertoire in this regard. Nevertheless, this distinction limits the extent to which the findings can be generalized to typical classroom conditions. Future research could address these limitations through larger-scale studies that include multiple schools and more extensive observation periods, and by combining prompted observations with naturalistic classroom observations to better capture the everyday enactment of SRL-supportive teaching.

Fourth, the potential influence of observer effects must be considered. Teachers may consciously demonstrate or conceal certain SRL strategies when being observed, which could affect the accuracy of the data. Longitudinal studies and repeated observations over time could help mitigate this effect, allowing researchers to examine how teachers’ SRL promotion evolves from explicit to implicit strategies, or from direct to indirect instruction, and how these changes relate to student needs and outcomes.

Finally, examining the effects of school-level factors on SRL implementation was beyond the scope of this study. Although some descriptive patterns suggested that school context may influence SRL-supportive practices more strongly than individual factors such as teacher experience or grade level, these observations should be interpreted with caution. Given the descriptive nature of the analyses and the small sample, no formal statistical testing was conducted, and the trends cannot be generalized beyond the current participants. Future research could use multi-level designs to explore how leadership, policy, and school culture shape the alignment between teachers’ knowledge, beliefs, and practices, providing more robust and generalizable insights into the role of school-level factors.

## 6. Conclusions

This study offers a novel, multi-method examination of how primary school teachers’ knowledge and beliefs about SRL may align with their classroom practices. By integrating video-based observations with teacher interviews, we captured both what teachers think and what teachers do regarding SRL. This approach addresses a gap in previous research which relied primarily on self-reports or quantitative measures.

Substantial variation was observed. Teachers focused mainly on metacognitive and motivational strategies, while cognitive strategies and indirect instructional dimensions, such as task value and cooperative learning, were underemphasized. There were indications of limited alignment between knowledge, beliefs, and practice, as well as limited self-efficacy, with school-level factors potentially shaping implementation across teachers within the same school.

These findings highlight the potential of multifaceted professional development that addresses knowledge gaps, misconceptions, and self-efficacy, while fostering collective reflective practices in school teams. Sustainable SRL promotion also requires school-wide strategies, including leadership support, collaboration, and engagement of students and parents. Overall, this study demonstrates that combining qualitative observations with interviews can provide unique insights into the complex interplay of what teachers think and what teachers do, offering practical guidance for enhancing SRL implementation in primary education.

## Figures and Tables

**Table 1:** Overview of participants and observed lessons.

Teacher	School	Gender	Age	Experience in Education	Experience in Current Grade	Grade	Experience in SRL *	Topic Observed Lessons	Instructional Context Observed Lessons	Adaptations to Everyday Teaching
**Teacher A**	S1	F	29	8	7	1	0	1: reading + mathematics 2: mathematics + spelling	1: teacher-centered teaching + silent work 2: teacher-centered teaching + working with small group	No No
**Teacher B**	S1	F	25	3	2	4	0	1: mathematics 2: group talks + mathematics + writing	1: silent work + teacher-centered teaching 2: silent work + teacher-centered teaching	No No
**Teacher C**	S2	F	32	9	9	6	1	1: individual student-teacher talk + mathematics 2: mathematics	1: working with small group + silent work 2: teacher-centered teaching + silent work	Yes No
**Teacher D**	S2	F	46	24	11	1	0	1: spelling 2: learning centers	1: teacher-centered teaching + silent work 2: task instruction + silent work	No No
**Teacher E**	S2	F	31	10	7	4	1	1: mathematics 2: mathematics	1: teacher-centered teaching + silent work 2: group work + teacher-centered teaching	No No
**Teacher F**	S2	M	35	14	4	3	2	1: painting 2: SRL practices	1: teacher-centered teaching + silent work 2: task instruction + silent work	Yes No
**Teacher G**	S3	F	27	3	1	6	0	1: mathematics 2: spelling	1: teacher-centered teaching + silent work 2: teacher-centered teaching + silent work	No No
**Teacher H**	S4	F	27	7	5	4	1	1: spelling + mathematics 2: language instruction	1: teacher-centered teaching + silent work 2: teacher-centered teaching + group work	No Yes

* Experience in SRL: 0 = No experience; 1 = Some experience (e.g., read a book, attended a short training); 2 = Extensive experience (e.g., participation in an intensive professional development program).

**Table 2:** Indirect instruction across six dimensions (average levels per teacher).

	Cooperative Learning	Constructivist Learning	Self-Determination	Value	Success Expectation	Student Support
**Mean**	1.81	2.56	2.55	1.81	2.77	3.39
**Teacher A**	1.50	2.00	2.20	1.13	2.50	3.13
**Teacher B**	1.33	2.00	3.00	1.38	2.75	3.25
**Teacher C**	1.00	2.25	2.70	1.75	2.75	3.25
**Teacher D**	2.17	2.75	3.30	2.50	3.50	3.75
**Teacher E**	2.83	2.87	2.70	1.88	2.50	3.25
**Teacher F**	1.17	2.50	1.40	2.00	2.25	3.25
**Teacher G**	1.17	3.00	2.20	1.25	2.75	3.50
**Teacher H**	3.33	3.13	2.90	2.63	3.13	3.75

**Table 3:** Alignment between teachers’ reported content knowledge and observed strategy implementation Green = high alignment; Orange = partial alignment; Blue = low alignment.

Teacher	What They Think (CK: Type of Strategy) *			What They Do (Observed Strategy Emphasis) **		
	Cognitive	Metacognitive	Motivational	Cognitive	Metacognitive	Motivational
**A**	/	+++	++	L	H	M
**B**	/	+++	++	L	M	L
**C**	+	+++	++	L	L	M
**D**	/	++	+++	M	M	H
**E**	+	+++	++	H	M	L
**F**	/	+++	++	L	L	L
**G**	+	+++	++	M	/	L
**H**	/	+++	++	M	M	H

* The symbol/denotes that the strategy type was not specified. The symbols +, ++, and +++ indicate increasing frequency, with +++ representing the highest. ** The symbol/denotes that this type of strategy was not observed. The symbols H, M, and L indicate the level of emphasis on that strategy, with H representing the highest emphasis, M medium emphasis, and L low emphasis.

**Table 4:** Alignment between the teachers’ reported and observed modes of direct instruction. Green = high alignment; Blue = low alignment.

Teacher	What They Think (PCK: Mode of Direct Instruction)		What They Do (Observed Mode of Direct Instruction)	
	Implicit	Explicit	Implicit	Explicit
**A**	x		x	
**B**	x			x
**C**		x		x
**D**	No references to direct instruction			x
**E**		x		x
**F**		x	x	
**G**		x	x	
**H**	x		x	

**Table 5:** Alignment between teachers’ reported knowledge and observed indirect instruction. Orange = partial alignment; Blue = low alignment.

Teacher	What They Think (PCK: Indirect Instruction) *				What They Do (Observed Indirect Instruction) **				
	Cooperative Learning	Self-Determination	Value	Succes Expectation	Cooperative Learning	Constructivist Learning	Self-Determination	Value	Student Support
**A**		1		2				L	H
**B**	2	1			L		H		
**C**		1		2	L				H
**D**		1		2	L				H
**E**		1		2		H		L	
**F**		1	2				L		H
**G**		1		2	L				H
**H**		1	2		L				H

* The two most frequently mentioned dimensions from the interviews are indicated, with 1 denoting the most frequently mentioned and 2 the second most frequently mentioned. ** H refers to the dimension with the highest score in the observed lessons, and L to the dimension with the lowest score.

## Data Availability

Data cannot be shared openly but are available on request from authors.

## Abbreviations

The following abbreviations are used in this manuscript:

SRL	Self-regulated learning
ATES	Assessing how Teachers Enhance Self-regulated learning
CK-SRL	Content knowledge about SRL
PCK-SRL	Pedagogical content knowledge about SRL

## Appendix A

**Table A1:** Constructs and sample items for the high-inference coding of teachers’ indirect promotion.

Construct (Number of Items)	Sample Item
Self-determination (4)	The teacher allows the students to take responsibility for structuring their learning by giving them some decision-making freedom.
Value (3)	Learning is integrated with a real-life context.
Success expectation (4)	The teacher allows the students to choose between different tasks (e.g., alternatives and/or difficulty levels).
Cooperative learning (3)	The teacher ensures that the students work together cooperatively and intervenes if necessary.
Student support (4)	The teacher shows the students that they take them seriously by asking positive questions. *
Constructivist learning (3)	The teacher integrates new knowledge in a meaningful context and/or introduces new knowledge by creating a cognitive conflict. *

* Based on the research of Rosenthal et al. (2024), we extended the ATES instrument with two constructs.

## Appendix B

**Table A2:** Overview of coding categories.

Code	Origin	Source
Definition of SRL		
**Cognitive strategies**	Deductive	(Boekaerts, 1999; Dignath et al., 2022)
- Repetition strategies		
- Elaborative strategies		
- Organizational strategies		
**Metacognitive strategies**	Deductive	(Boekaerts, 1999; Dignath et al., 2022)
- Planning strategies		
- Monitoring strategies		
- Evaluation strategies		
**Motivational strategies**	Deductive	(Boekaerts, 1999; Dignath et al., 2022)
- Increasing the task value		
- Learning environment/environmental control		
- Use of social resources		
- Self-esteem stabilization		
- Volition/effort control		
**Misconceptions about SRL**	Inductive	
- SRL = learning independently		
- SRL = focusing on wellbeing		
- SRL = offering less structure		
**SRL implementation in the classroom**		
**Physical elements**	Inductive	
- Providing tools & resources		
- Organizing the classroom and classroom layout		
**Direct instruction**	Deductive	(Dignath et al., 2022)
- Explicit		
- Implicit		
**Indirect instruction**	Deductive	(Dignath et al., 2022)
- Cooperative learning	Deductive	(Dignath et al., 2022)
- Constructivist learning	Deductive	(Dignath et al., 2022)
○ *Activating prior knowledge*		
○ *Integrating new knowledge into meaningful contexts*		
○ *Complex, open-ended problems*		
○ *Asking open-ended questions*		
- Self-determination	Deductive	(Dignath et al., 2022)
○ *Freedom of decision-making for students*		
○ *Balance between self-directed and teacher-directed learning*		
○ *Open forms of teaching and learning*		
○ *Offering choices*		
○ *Self-evaluation*		
- Transfer	Deductive	(Dignath et al., 2022)
○ *Real-life context*		
○ *Diverse contexts and perspectives*		
○ *Value of learning content*		
○ *Learning material from everyday life*		
- Control/expectation	Deductive	(Rosenthal et al., 2024)
○ *Choice of task*		
○ *Competence-oriented feedback*		
○ *Clear, targeted instructions*		
○ *Providing resources*		
- Student support	Deductive	(Rosenthal et al., 2024)
○ *Taking students seriously*		
○ *Supporting students when ridiculed, ashamed, or embarrassed*		
○ *Patience with students’ mistakes*		
○ *Mistakes = learning opportunities*		
**Teaching approach**	Inductive	
- Emphasizing differentiation		
- Gradual introduction into classroom practice		
- Teacher-directed		
- Use of textbooks		
- Use of ICT		
**Monitoring and follow-up of students**	Inductive	
- SRL skills/learning process		
- Subject-specific performance		
**Challenges in classroom practice**	Inductive	
- Mult-tasking		
- Extra workload		
- Time investment		
**Teacher characteristics**		
Positive beliefs	Deductive	(Lombaerts et al., 2009)
Uncertainty/low self-efficacy	Deductive	(De Smul et al., 2018)
Self-reflection	Inductive	
**Student characteristics**		
Student age	Inductive	
Student ability level	Inductive	
Learning difficulties/disorders	Inductive	
Home situation/parents	Inductive	
**Support needs**		
Practical tools	Inductive	
Theoretical support	Inductive	
Visits to other teachers/schools	Inductive	
In-class support	Inductive	
Training or professional development initiatives	Inductive	
**School level**		
School vision on SRL	Inductive	
Collaboration and support at school level	Inductive	
Learning trajectory/continuity	Inductive	
Role of school leader	Inductive	
